# Subacute invasive pulmonary aspergillosis after chemoradiotherapy for lung cancer

**DOI:** 10.1002/rcr2.523

**Published:** 2020-02-03

**Authors:** Hirofumi Watanabe, Toshihiro Shirai, Mika Saigusa, Kazuhiro Asada, Kazumori Arai

**Affiliations:** ^1^ Department of Respiratory Medicine Shizuoka General Hospital Shizuoka Japan; ^2^ Department of Pathology Shizuoka General Hospital Shizuoka Japan

**Keywords:** Chemoradiotherapy, invasive pulmonary aspergillosis, lung cancer, subacute

## Abstract

Subacute invasive pulmonary aspergillosis (SIPA), a rapidly progressive fungal infection of less than three months arising from pre‐existing lung lesions, generally afflicts moderately immunocompromised patients. We herein report the case of a 69‐year‐old man who developed SIPA following chemoradiotherapy for lung cancer and treated with antifungal therapy. He presented with fever, and computed tomography revealed a cavity with surrounding consolidation. The cavity itself had been considered as the primary tumour treated by chemoradiotherapy. Bronchoalveolar lavage by bronchoscopy performed at admission identified *Aspergillus fumigatus*; no other pathogens or malignant cells were observed. Owing to the worsening of symptoms and inflammation despite micafungin administration, the treatment was changed to liposomal amphotericin B with voriconazole, which led to clinical improvement. In addition to cancer recurrence and bacterial infection, fungal infection should also be considered in patients undergoing chemoradiotherapy for lung cancer with deteriorating imaging findings and symptoms. In intractable cases, multiple antifungal drugs are effective.

## Graphical abstract

Subacute invasive pulmonary aspergillosis (SIPA), a rapidly progressive fungal infection of less than three months arising from pre‐existing lung lesions, generally afflicts moderately immunocompromised patients. We herein report the case of a 69‐year‐old man who developed SIPA following chemoradiotherapy for lung cancer and treated with multiple antifungal therapy.

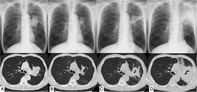

## Introduction

Pulmonary aspergillosis is an intractable fungal infection that develops in immunocompromised patients with pre‐existing structural damage such as emphysema, cysts, and old pulmonary tuberculosis and those treated for lung cancer 1, 2. Subacute invasive pulmonary aspergillosis (SIPA), which is sometimes categorized as a chronic pulmonary aspergillosis (CPA), is a rapidly progressive condition that should be treated as invasive pulmonary aspergillosis 1. We herein report a patient who was diagnosed with SIPA by bronchoscopy after undergoing chemoradiotherapy for lung cancer. The patient was treated with multiple agents as a single antifungal agent was ineffective.

## Case Report

**Figure 1:**
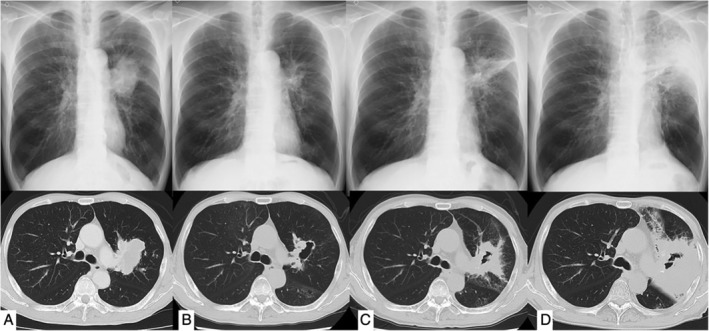
(A) Computed tomography and X‐ray images before treatment showing lung tumour in left lung. (B) After chemoradiotherapy, primary lesion is smaller, forming a cavity. (C) On admission, note the thickening of the cavity wall and consolidation around the cavity. (D) Images on the seventh day after admission showing worsening of the findings despite the initiation of micafungin treatment.

A 69‐year‐old male presented to the hospital with a one‐month history of fever and cough. Four months before admission, he had completed concurrent chemoradiotherapy for stage IIIB (cT4N2M0) squamous cell carcinoma of the lung (Fig. 1A). Regimen of chemotherapy consisted of carboplatin AUC2 and paclitaxel 40 mg/m^2^ on day 1 and repeated every week. He received six cycles of chemotherapy and a total of 60 Gy radiation with three‐dimensional conformal radiation therapy. No complications were encountered during chemoradiotherapy including neutropenia. After chemoradiotherapy, the inside of the tumour was necrotic and became a cavity (Fig. 1B). Two months before admission, he developed radiation pneumonitis and was receiving oral prednisolone. Bronchoscopy performed before the initiation of prednisolone did not reveal any pathogens. At first, he was administered prednisolone 20 mg/day and reduced amount of prednisolone every two weeks. He smoked for 49 pack‐years before diagnosis of lung cancer, and his medical history included chronic obstructive pulmonary disease (COPD), which was treated with tiotropium bromide hydrate 3.124 μg/olodaterol hydrochloride inhaler 2.736 μg. He neither had pets nor a hot tub and was unemployed. Before admission, the patient's symptoms did not improve despite oral levofloxacin 500 mg/day administered for two weeks; he was on oral prednisolone (5 mg/day).

**Figure 2:**
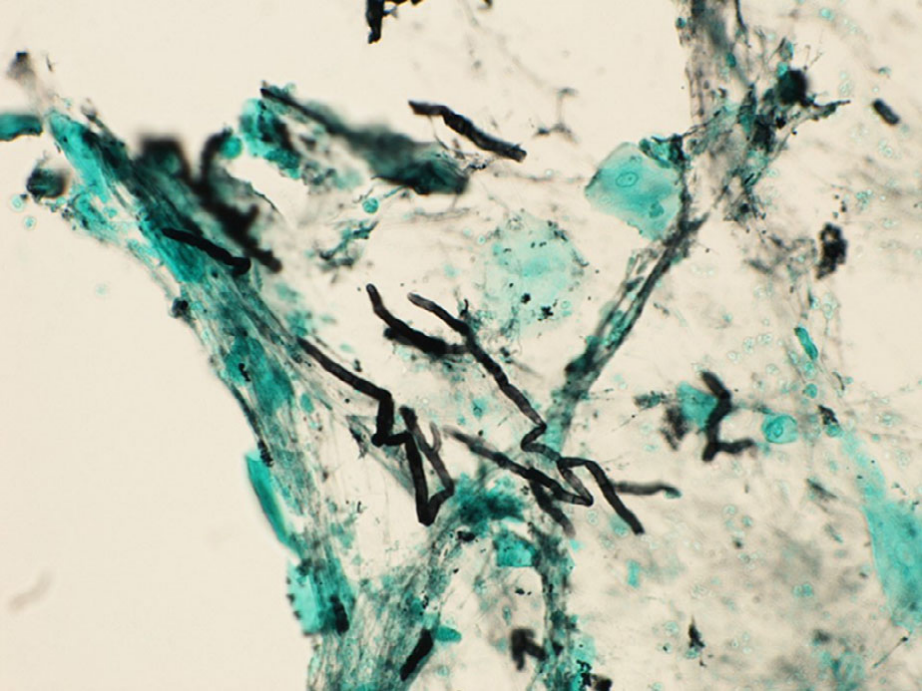
Bronchoalveolar lavage fluid stained by Grocott showing the presence of fungi.

On examination, he was 157.8 cm in height and weighed 48.5 kg. He was ill‐appearing and febrile with a temperature of 38.2°C. His other vital signs were as follows: blood pressure, 90/63 mmHg; pulse, 103/min; and respiratory rate, 20/min with 93% O_2_ saturation on room air. Breath sounds were reduced on the left side. The laboratory results on admission were as follows: white blood cells, 5800/mm^3^ with 85.2% neutrophils and 7.7% lymphocytes; haemoglobin, 9.3 g/dL; albumin, 2.8 g/dL; and C‐reactive protein, 14.4 mg/dL. β‐D‐glucan, a fungal cell wall constituent, level was normal (<11.0 pg/mL). The serum galactomannan antigen was negative, whereas the immunoglobulin G antibody against *Aspergillus* was positive. Chest X‐ray and computed tomography images showed a cavity with surrounding consolidation with a thick wall (Fig. 1C). Bronchoscopy was performed on the first day of admission for bronchoalveolar lavage; biopsy was not performed due to the concern regarding bleeding. Initially, intravenous tazobactam/piperacillin 13.5 mg/day was administered; however, following the detection of fungi by staining of the bronchoalveolar lavage fluid in the absence of malignant cells (Fig. 2), micafungin (150 mg/day) was started on the fourth day for the presumptive diagnosis of SIPA. As the patient's condition worsened over time, the micafungin dose was increased to 300 mg/day on the fifth day. His condition did not improve (Fig. 1D), and the antifungal agent was changed to liposomal amphotericin B (L‐AMB) at 150 mg/day on the ninth day. However, fever and cough did not improve, and intravenous voriconazole (540 mg/day as the loading dose followed by 360 mg/day as the maintenance dose) was added on the 15th day. *Aspergillus fumigatus* was detected later in culture, and definitive diagnosis of SIPA was made. The patient recovered gradually. L‐AMB was used for 38 days, and voriconazole was changed from intravenous to oral use (300 mg/day) on day 40 after admission; he was discharged on day 51 after admission. The trough concentrations of voriconazole were 1.39 and 3.57 mg/mL on days 23 and 47, respectively.

## Discussion

We herein presented a patient who developed SIPA at the site of treatment following chemoradiotherapy, which required multiple antifungal agents for the clinical improvement of the patient.

Aspergillosis following treatment for lung cancer is often reported in patients after surgery, sometimes after radiotherapy, but they are basically chronic progression in months or years 1. This case developed and deteriorated in a short time of about one month. Before the treatment with prednisolone for radiation pneumonitis, no fungi were detected by bronchoscopy. Owing to the presence of cavity lesion after chemoradiotherapy, the history of COPD and the steroid usage for radiation pneumonitis, *A. fumigatus* easily had colonized, which might explain the relatively fast deterioration of the clinical condition 2, 3. Antibody against *Aspergillus* may be positive in SIPA 1. Galactomannan and β‐D‐glucan, both fungal cell wall constituent, are useful as diagnostic tests of pulmonary aspergillosis, especially for haematological patients with invasive pulmonary aspergillosis 2. The current patient was finally diagnosed with SIPA based on the detection of *A. fumigatus* in the bronchoalveolar lavage fluid culture obtained from the lesion.

Echinocandins represented by micafungin, triazoles represented by voriconazole, and polyenes represented by amphotericin B are used to treat subacute invasive or invasive aspergillosis. Micafungin was initiated instead of voriconazole, the recommended first choice, due to its relatively few side effects as the patient's general condition was not good. The micafungin dose was increased from 150 to 300 mg/day, but the imaging findings and inflammation worsened. Therefore, the treatment was changed to L‐AMB. However, voriconazole was added because the effect of L‐AMB was poor. Studies previously suggested that combination therapy, especially that of echinocandins with voriconazole or amphotericin B, was useful in SIPA. Additionally, polyenes and triazole were reported to antagonize each other in vitro 4, albeit it remains unclear whether this should be a concern in vivo in humans 5. The current patient was switched from micafungin to L‐AMB; however, it remains possible that continuation of micafungin with the addition of L‐AMB or voriconazole might have been appropriate.

Distinguishing pulmonary aspergillosis from other infections or cancer recurrence is challenging if it develops at the treatment site forming cavity following radiotherapy. Though SIPA is categorized as CPA, it is life‐threatening disease, such as invasive pulmonary aspergillosis 1. Therefore, patients with suspicious SIPA should be evaluated by bronchoscopy and treated immediately. In intractable cases, multiple antifungal drugs are effective.

### Disclosure Statement

Appropriate written informed consent was obtained for publication of this case report and accompanying images.
